# The Relationship Between Paternal Preconception Obesity and Health Behaviors and Childhood Obesity: Protocol for a Systematic Review

**DOI:** 10.2196/31254

**Published:** 2021-12-02

**Authors:** Marie-Eve Laforest, Stephanie Ward, Liette-Andrée Landry, Fabrice Mobetty

**Affiliations:** 1 École de Science Infirmière Faculté des Sciences de la Santé et des Services Communautaires Université de Moncton Moncton, NB Canada; 2 École des Sciences des Aliments, de Nutrition et d’Etudes Familiales Faculté des Sciences de la Santé et des Services Communautaires Université de Moncton Moncton, NB Canada; 3 École de Science Infirmière Faculté des Sciences de la Santé et des Services Communautaires Université de Moncton, Campus de Shippagan Shippagan, NB Canada; 4 Département de Nutrition Faculté de Médecine Université de Montréal Montreal, QC Canada

**Keywords:** childhood obesity, preconception, paternal obesity, health behaviours, obesity, public health, children, adolescents, body weight, parenting, health behaviors

## Abstract

**Background:**

Childhood obesity is a global public health concern and is a priority for researchers and policy makers. To overcome the epidemic of obesity, influencing factors throughout the life span need to be addressed, including those in the preconception period. A better understanding of the association between paternal preconception factors and childhood obesity is important for public health interventions.

**Objective:**

This systematic review will examine the relationship between paternal preconception obesity and health behaviors and their offspring’s overweight or obesity.

**Methods:**

Peer-reviewed quantitative studies and grey literature that report associations between paternal preconception obesity and health behaviors—such as smoking, exercise, and eating habits—and childhood overweight and obesity will be identified through a computerized literature search in 7 databases. The quality of each study will be assessed using the Quality Assessment Tool for Quantitative Studies. Characteristics of the included studies will be reported, and relevant findings from each paternal preconception exposure will be narratively synthesized. This review will follow the PRISMA (Preferred Reporting Items for Systematic reviews and Meta-Analyses) 2020 guidelines.

**Results:**

This systematic review is anticipated to begin in December 2021 and be completed by the end of August 2022.

**Conclusions:**

This systematic review will contribute to a better understanding of the relationship between preconception paternal exposures and their offspring’s overweight or obesity. Findings will help support health professionals working with prospective parents to educate fathers on the benefits of improving their weight and health behaviors during the preconception period.

**International Registered Report Identifier (IRRID):**

PRR1-10.2196/31254

## Introduction

Childhood obesity is a widely acknowledged health concern whose prevalence is increasing rapidly at the international level. According to the World Health Organization, childhood obesity is defined as an abnormal or excessive accumulation of body fat that can be harmful to health [1]. In 2016, the global prevalence of childhood obesity was assessed at 18% [2]. At that time, it was estimated that approximately 340 million children and adolescents between 5 and 19 years of age were obese or overweight, which represents a considerable increase over the last 30 years [2].

The high prevalence of childhood obesity not only poses a considerable challenge to the health care system in the future, but it can also affect children's short- and long-term health and well-being. For example, children who are obese have an increased risk of cardiovascular disease [3], including coronary heart disease and atherosclerosis, gastrointestinal disease [4], diabetes, and even some cancers in adulthood [5]. In addition to these long-term serious physical health conditions, obesity can also lead to various psychological, social, and educational consequences in childhood [6-9]. Therefore, global calls for action have been made to halt the rise in childhood obesity [1].

Various risk factors are associated with childhood obesity, making this public health issue a complex one to solve. Research has recognized that risk factors can be behavioral, biological, environmental, and societal [10-13]. According to the Commission on Ending Childhood Obesity [1], risk factors throughout childhood need to be addressed, beginning with preconception. In fact, there is growing evidence that an individual's genetic makeup is important in determining the risk of obesity [8]. Thus, it is recommended that health professionals address modifiable risk factors of both parents before conception [14-16]. For example, according to Barker et al [17], children's growth, development, and long-term health can be shaped by parental nutritional status before conception.

Studies examining preconception risk factors of childhood obesity have mainly focused on mothers. This focus on women has also contributed to gender bias, suggesting that women bear the sole responsibility for their child's health outcomes [18,19]. Nevertheless, growing evidence from human and animal studies suggests that preconception paternal risk factors could also play an important role in the health and development of their offspring [13,14,16,18-21]. For example, Braun et al [22] found that various paternal behavioral risk factors before conception, such as stress and diet, were associated with some diseases and obesity in children. Similarly, Mejia-Lancheros et al's [23] prospective cohort study found a positive association between paternal smoking during preconception or the early gestational period and childhood obesity at 5 years of age. Comparably, a longitudinal study by Northstone et al [24] found a positive association between fathers who started smoking before the age of 11 years and their son’s BMI in adolescence. Transgenerational effects of smoking on adolescents’ body composition have also been studied. Dougan et al [25] found that grandpaternal smoking was positively associated with their granddaughter’s obesity or overweight at 12 years of age but not with their grandson’s weight.

In addition to behavioral risk factors, paternal preconception weight has also been linked to obesity or overweight in children [26-28]. For example, Rath et al [28] found that 14-year-old adolescents were three times more likely to be obese if their father was overweight before conception compared to those who had fathers who had a healthy weight before conception. Furthermore, the risk of obesity at 14 and 22 years old quadrupled for children whose father was obese before conception [28]. Similar to Dougan et al’s [25] study, Jääskeläinen et al [27] found that paternal overweight or obesity preconception was a stronger predictor of overweight or obesity among daughters than sons.

The link between paternal preconception weight and behaviors and childhood obesity may be partially explained by epigenetics. Specifically, epigenetics involves the transmission or modification of gene expression, which is inherently responsible for health and disease pathogenesis [18,19,29-31]. It has been reported that gene expression could be influenced by adiposity [32] and various health behaviors, such as tobacco use, physical activity, and unhealthy eating [33]. Since children inherit a complete set of genes from each parent, paternal weight and health behaviors may modify fathers’ gene expression, which can be passed down to their offspring and future generations. Although some researchers claim that genetics plays only a small role in childhood obesity [8,34], others argue that parents’ health at the time of conception is critical in shaping the health of their future child and therefore deserves particular consideration [20,35].

While most systematic reviews have reported on the impact of paternal BMI on child health outcomes [36], paternal risk factors and offspring cardiometabolic disease [16], and paternal BMI and childhood obesity [37], none have looked at preconception paternal obesity and health behaviors, such as diet, exercise, and smoking, and their association with childhood obesity. A better understanding of this relationship would help support the implementation of interventions before conception and help address the epidemic of childhood obesity. This evidence is also needed to support public health action throughout the preconception period.

This systematic review will summarize peer-reviewed studies and grey literature publications that have examined how paternal preconception obesity and health behaviors are associated with childhood obesity. Specifically, this review will examine how fathers’ obesity and health-related behaviors such as smoking, exercise, and eating habits before conception are associated with their offspring’s weight status. The main objective of this review will be to identify the potential importance of preconception health and behaviors of fathers on their offspring’s health and suggest avenues for future research.

## Methods

### Design

This systematic review will follow the updated guidelines from the PRISMA (Preferred Reporting Items for Systematic reviews and Meta-Analyses) 2020 statement [38]. The 27-item checklist from the PRISMA 2020 statement will be used to ensure full transparency and completeness of the reporting, particularly related to the methods used to identify, select, appraise, and synthesize the included studies [39].

### Eligibility Criteria

Studies will be included in this systematic review if they meet the following criteria: (1) they assess the unique contribution of paternal preconception obesity or health behaviors to children's weight status, (2) they focus on one or more paternal health behaviors (ie, smoking, exercise, and eating habits) or overweight/obesity before conception, (3) they focus solely on the weight status of children <18 years of age, and (4) they are published in either English or French. Studies that include secondary analyses that meet the above criteria will also be included. Studies that focus on paternal prenatal (after conception) health or behaviors, or those that include fathers with complex health conditions (eg, diabetes, cardiovascular disease), will be excluded from this review.

### Types of Studies

Peer-reviewed, quantitative studies will be included in this systematic review. Due to the nature of this systematic review’s objectives, prospective and retrospective cohort studies will be included to assess the relationship between paternal preconception obesity and health behaviors and childhood overweight/obesity. Although unlikely, experimental study designs (randomized controlled trials, clinical control trials, pre-post designs) will also be considered if they meet the inclusion criteria. To ensure all relevant literature is identified, reference lists of any previous systematic, scoping, or narrative reviews will also be checked. Grey literature (eg, dissertations, theses, reports) will also be included in this review, considering the novelty of this topic and the potentially limited number of relevant publications. Qualitative and animal studies will be excluded from this review.

### Population and Exposure of Interest

This review will focus on males who may or may not have been actively trying to conceive. Objectively and subjectively measured paternal preconception overweight/obesity and health behaviors will be the primary exposures included in this review. Health behaviors will include smoking (ie, cigarettes, tobacco, vaping), exercise (ie, physical activity, sedentary behavior), and eating habits (ie, dieting, dietary intake, food intake, behaviors), as these have been shown to influence gene expression in human or animal studies.

### Outcome of Interest

Objectively or subjectively measured childhood obesity will be the primary outcome of this systematic review. Childhood will encompass any child <18 years of age. Childhood obesity will be defined as any child reported to have overweight or obesity, based on the method of measurement’s guidelines (eg, World Health Organization, Centers for Disease Control and Prevention, BMI). All body composition or adiposity measurements will be included, such as BMI, waist circumference, waist to height ratio, skin folds, bioelectrical impedance analysis, and dual-energy X-ray absorptiometry (DXA).

### Search Methods

The search strategy used for this review will be developed in collaboration with an experienced research librarian. Relevant studies will be identified through a computerized search in the following databases: Cochrane, PubMed, EBSCO Host (CINAHL, APA PsycINFO), ProQuest, Scopus (Science Direct), and Google Scholar. A specific search strategy will be formulated in PubMed and adapted for each of the above databases (Table 1). Reference lists of retained studies will also be searched to ensure that all relevant studies have been identified. All articles that will have emerged from the computerized search will be exported to Mendeley, and any duplicates will be removed.

**Table 1:** Strategy search sample for PubMed.

Concepts	Mesh and keywords
Paternal	(“Fathers”[Mesh] OR “Paternal Inheritance”[Mesh] OR “Paternal Behavior”[Mesh] OR “Paternal Exposure”[Mesh] OR “Paternal Age”[Mesh] OR “Father-Child Relations”[Mesh] OR father*[Title/Abstract] OR paternal[Title/Abstract])
Preconception	(“Preconception Care”[Mesh] OR “Fertilization”[Mesh] OR “Posthumous Conception”[Mesh] OR “Prenatal Care”[Mesh] OR “Prenatal Exposure Delayed Effects”[Mesh] OR preconception[Title/Abstract] OR pre-conception[Title/Abstract] OR fertiliz*[Title/Abstract])
Health habits and obesity	(“Obesity”[Mesh] OR “Overweight”[Mesh] OR “Body Composition”[Mesh] OR “Body Weight”[Mesh] OR “Intra-Abdominal Fat”[Mesh] OR “Body Fat Distribution”[Mesh] OR “Adipose Tissue”[Mesh] OR “Life Style”[Mesh] OR “Healthy Lifestyle”[Mesh] OR “Smoking”[Mesh] OR “Tobacco Products”[Mesh] OR “Smokers”[Mesh] OR “E-Cigarette Vapor”[Mesh] OR “Vaping”[Mesh] OR “Cigarette Smoking”[Mesh] OR “Nutritional Status”[Mesh] OR “Diet Therapy”[Mesh] OR “diet therapy” [Subheading] OR “Eating”[Mesh] OR “Exercise”[Mesh] OR “Sedentary Behavior”[Mesh] OR “Sports”[Mesh] OR “Physical Exertion”[Mesh] OR “Risk Factors”[Mesh] OR “Health Risk Behaviors”[Mesh] OR obese[Title/Abstract] OR obesity[Title/Abstract] OR “body composition” [Title/Abstract] OR “body weight”[Title/Abstract] OR “body fat”[Title/Abstract] OR “Intra-abdominal fat”[Title/Abstract] OR “adipose tissue”[Title/Abstract] OR “life style”[Title/Abstract] OR smoking[Title/Abstract] OR tobacco[Title/Abstract] OR smoker*[Title/Abstract] OR “e-cigarette” [Title/Abstract] OR vaping[Title/Abstract] OR “nutritional status”[Title/Abstract] OR diet[Title/Abstract] OR exercise[Title/Abstract] OR sedentary[Title/Abstract] OR “physical activity”[Title/Abstract])
Childhood obesity	(“Pediatric Obesity”[Mesh] OR (“Child”[Mesh] AND “Obesity”[Mesh]) OR “pediatric obesity“ [Title/Abstract] OR “child obesity”[Title/Abstract] OR (“Child”[Mesh] AND “body composition”[Title/Abstract]) OR (“Child”[Mesh] AND “Adiposity*”[Mesh]) OR (“Child”[Mesh] AND “Body Mass Index”[Mesh]) OR (“Child”[Mesh] AND “Waist Circumference”[Mesh]) OR (“Child”[Mesh] AND “Waist-Height Ratio”[Mesh]) OR (“Child”[Mesh] AND “DXA” [Title/Abstract]) OR (“Child”[Mesh] AND “skin fold” [Title/Abstract]))

### Selection of Studies

All titles and abstracts that will emerge from the computerized search will be independently assessed for relevancy by at least two investigators. Half of the titles and abstracts will be assessed by MEL and LAL, and the other half will be assessed by SW and FM. If there is a disagreement between the two investigators, the full text will be verified by a third investigator. The full text of all remaining articles will be read by at least two investigators, who will independently assess them against the eligibility criteria (MEL and LAL will assess half of the articles and SW and FM will assess the other half). In the case of a disagreement, the full text will be read by a third investigator. All studies that do not meet eligibility criteria will be excluded. In cases where the full text of an article cannot be accessed, efforts will be made to contact the corresponding author of that article. As per the PRISMA 2020 guidelines, a flow diagram depicting how articles were identified, screened, and included in the systematic review will be constructed. The full text of all remaining relevant studies will be kept in a folder shared between the investigators in Mendeley.

### Data Extraction and Management

Two independent investigators will enter data from each relevant study into a spreadsheet software such as Excel (MEL and LAL will enter data from half of the studies, and SW and FM will enter data from the remaining half). Extraction issues and missing data problems will be resolved through discussion among the four investigators. The following variables will be extracted from each article: (1) characteristics of the study (ie, author names and publication date, title of the study, country/location of origin), (2) the study’s goals or objectives, (3) type of study (ie, design, length of follow up), (4) characteristics of the population (ie, child and paternal age, sample size, time of preconception, ethnicity), (5) exposure variables (ie, paternal weight and health behaviors) of interest, (6) methods of assessment or tools for both paternal and childhood obesity variables, (7) methods of analysis, and (8) main results and study limitations.

### Quality Assessment

All included studies will be assessed for quality using the Quality Assessment Tool for Quantitative Studies. This validated tool was developed to improve the quality of systematic review reporting, particularly in public health contexts [38]. This tool involves giving a strong, moderate, or weak rating for 8 indicators, including selection bias, study design, confounders, blinding, data collection methods, withdrawals and dropouts, intervention integrity, and analysis. These ratings are then used to provide an overall methodological rating for the article in question. Compared to other tools that were mainly developed to assess the quality of experimental studies, the Quality Assessment Tool for Quantitative Studies can evaluate the quality of observational studies. For this study, two investigators will independently assess the quality of each of the studies included in the systematic review (MEL and LAL will assess half of the included studies, and SW and FM will assess the remaining half). In the case of a disagreement, a third investigator will evaluate the quality of the study.

### Data Analysis and Reporting

Since it is anticipated that there will be considerable heterogeneity in study exposure, methods, and measurement tools, data will be narratively synthesized. A descriptive summary of the studies’ characteristics will first be presented. This summary will be supported by a table presenting all extracted data from the included studies, including their methodological quality assessment. The outcomes of this review will be presented and discussed in line with each paternal preconception exposure. If possible, results will also be stratified by the offspring’s age—that is, infants and toddlers (birth to 2 years), preschoolers (3-5 years), school-age children (6-10 years), and preadolescents and adolescents (11-17 years). The overall strength of evidence will be determined by the strength of the methodological assessment of each study, the number of studies included, and the homogeneity of the findings across the studies. Implications for childhood obesity prevention and recommendations for future research avenues will be discussed.

## Results

This systematic review is anticipated to begin in December 2021. Study selection and data extraction will begin in February of 2022, and quality assessment and data synthesis will begin in April of 2022. The review is expected to be completed by the end of August 2022.

## Discussion

### Overview

Over the past decades, childhood obesity rates have increased dramatically across the globe. Although obesity is a multifaceted disease that is influenced by environmental, behavioral, biological, and genetic factors, it is well recognized that early intervention is necessary to prevent the onset of childhood obesity and its short- and long-term health consequences. Emerging research in epigenetics has suggested that the preconception period may be critical for obesity prevention. Some animal and human studies have supported this notion by reporting strong associations between maternal preconception risk factors and their offspring’s obesity. However, evidence on how paternal preconception risk factors are associated with childhood obesity has never been synthesized.

Given the urgency of addressing childhood obesity and the possible role of epigenetics in the onset of this chronic condition, identifying potential parental preconception risk factors may be one strategy to intervene at the earliest possible time. Although mothers have been the target of previous preconception public health interventions, fathers have been relatively dismissed. Therefore, this systematic review will provide valuable information on whether paternal preconception obesity and health behaviors are linked to their offspring’s obesity and body weight. Findings from this review may be used as a tool by health care professionals working with prospective parents to educate fathers on the benefits of improving their weight status and health behaviors alongside their partner during the preconception period.

### Strengths and Limitations of the Review

This systematic review will be the first to synthesize evidence related to the association between paternal preconception obesity and health behaviors and their offspring’s obesity. One of the main strengths of this systematic review is the breadth of health behaviors that will be assessed, allowing for a more comprehensive overview of the topic. Other strengths of this review include using a comprehensive search strategy in 7 different databases, using a validated tool to assess observational-type studies, including studies published in either English or French, and not limiting the publication period.

Since epigenetics is a relatively new field of research, it is possible that only a small number of studies will be included in this review. This limitation must be acknowledged, as it could limit the strength of the overall conclusions.

### Dissemination

Any changes made to this protocol will be reported, with justification, in the final review. Findings from this systematic review will be disseminated through traditional approaches, including scientific peer-reviewed publications and conferences. Nontraditional methods of dissemination will also be used, including fact sheets for primary care providers (eg, family physicians, nurses, fertility specialists), public health nurses and dietitians, patients, and the general public.

## Abbreviations

DXA: dual-energy X-ray absorptiometry
PRISMA: Preferred Reporting Items for Systematic reviews and Meta-Analyses
